# Taking the heat or taking the temperature? A qualitative study of a large-scale exercise in seeking to measure for improvement, not blame

**DOI:** 10.1016/j.socscimed.2017.12.033

**Published:** 2018-02

**Authors:** Natalie Armstrong, Liz Brewster, Carolyn Tarrant, Ruth Dixon, Janet Willars, Maxine Power, Mary Dixon-Woods

**Affiliations:** a Department of Health Sciences, University of Leicester, Leicester, UK; b Lancaster Medical School, Lancaster University, Lancaster, UK; c Blavatnik School of Government, University of Oxford, Oxford, UK; d Department of Politics and International Relations, University of Oxford, Oxford, UK; e Haelo, Salford, UK; f The Healthcare Improvement Studies (THIS) Institute, University of Cambridge, Cambridge, UK

**Keywords:** United Kingdom, Patient safety, Measurement, Quality, Improvement, Qualitative research, Performance management, Harm-free care

## Abstract

Measurement of quality and safety has an important role in improving healthcare, but is susceptible to unintended consequences. One frequently made argument is that optimising the benefits from measurement requires controlling the risks of blame, but whether it is possible to do this remains unclear. We examined responses to a programme known as the NHS Safety Thermometer (NHS-ST). Measuring four common patient harms in diverse care settings with the goal of supporting local improvement, the programme explicitly eschews a role for blame.

The study design was ethnographic. We conducted 115 hours of observation across 19 care organisations and conducted 126 interviews with frontline staff, senior national leaders, experts in the four harms, and the NHS-ST programme leadership and development team. We also collected and analysed relevant documents.

The programme theory of the NHS-ST was based in a logic of measurement for improvement: the designers of the programme sought to avoid the appropriation of the data for any purpose other than supporting improvement. However, organisational participants - both at frontline and senior levels - were concerned that the NHS-ST functioned latently as a blame allocation device. These perceptions were influenced, first, by field-level logics of accountability and managerialism and, second, by specific features of the programme, including public reporting, financial incentives, and ambiguities about definitions that amplified the concerns. In consequence, organisational participants, while they identified some merits of the programme, tended to identify and categorise it as another example of performance management, rich in potential for blame.

These findings indicate that the search to optimise the benefits of measurement by controlling the risks of blame remains challenging. They further suggest that a well-intentioned programme theory, while necessary, may not be sufficient for achieving goals for improvement in healthcare systems dominated by institutional logics that run counter to the programme theory.

## Highlights

• Measurement for healthcare improvement is susceptible to unintended consequences.
• The NHS Safety Thermometer sought to optimise the benefits by disavowing blame.
• We examined responses to the NHS Safety Thermometer using ethnographic methods.
• Fear of blame often limited use of the tool as a way to measure for improvement.
• A sound programme theory can be challenged by competing institutional logics.

## 1 Introduction

Quality measurement that focuses on important processes and outcomes, including clinical care and patient experience, is often seen as an essential feature of well-functioning healthcare systems (Conway et al., 2013). Prominent uses of measurement include identifying variations in care between different organisations or practitioners, surfacing the factors associated with high performance, and supporting replication and scaling of apparently successful approaches (Bradley et al., 2012). Measurement is a defining characteristic of many quality improvement efforts, where techniques such as statistical process control and audit and feedback are routinely deployed to help practitioners monitor their local system performance and the responses of that system to improvement interventions (Portela et al., 2015, Ivers et al., 2012). Increasingly, measurement is also deployed in the context of performance management regimes and as an element of pay-for-performance schemes to address the demand for accountability and transparency that has become one of the central tropes of current thinking on governance and regulation in modern healthcare (Mukamel et al., 2014, Brewster et al., 2016). These differing goals of measurement embody a number of tensions, including those relating to the balance between stimulating improvement and provoking unintended consequences – such as gaming (manipulation of data to look good), effort substitution (focus on the things being measured to the exclusion of other important activities) (Kelman and Friedman, 2009), shrinkage of professional responsibility (narrow focus on the things being measured) and excessive bureaucratic burden associated with data collection and reporting (Chassin et al., 2010, Meyer et al., 2012). The question of whether it is possible to capture the potential benefits of measurement while minimising the risks is thus a critical one.

One frequently made argument is that optimising the yield from measurement (and, conversely, averting its unwanted effects) requires controlling the risks of *blame*. Solberg et al.'s much-cited article (Solberg et al., 1997) distinguishes *measurement for improvement* from *measurement for accountability,* proposing that the appropriation of locally collected data for external accountability purposes may thwart the goal of improvement. These authors argue that only when fear and blame are “out of the equation”, can everyone “concentrate on improvement rather than defensiveness” (p.138). Yet cultures of blame are pervasive in healthcare (Dekker and Hugh, 2014), resulting, Don Berwick argues, in measurement fostering fear and defensiveness rather than improved quality and safety:

*Any good foreman knows how clever a frightened work force can be. In fact, practically no system of measurement - at least none that measures people's performance - is robust enough to survive the fear of those who are measured […] The inspector says, "I will find you out if you are deficient." The subject replies, "I will therefore prove I am not deficient" - and seeks not understanding, but escape.* (Berwick, 1989:53)

It remains unclear, however, whether it is possible to design and operate measurement systems for improving healthcare quality that evade the apparently negative effects of blame: field studies of measurement of quality and safety have remained rare (Dixon-Woods et al., 2012).

We suggest one useful way of gaining clarity and analytic purchase is to understand “data for accountability” and “data for improvement” as two different logics. We propose, as a more general principle, that quality improvement (QI) efforts founded in a particular logic may be overwhelmed by institutional logics operating at the field level. We provide empirical support for this analysis using an ethnographic study of a large-scale data collection programme in England. Known as the NHS Safety Thermometer, the programme is of particular interest for our purposes because it explicitly embraced a principle of “data for improvement” that eschewed a role for blame.

### 1.1 Blame

It is useful to begin by acknowledging that, though blame is widely discussed in the healthcare literature and at policy level, for example in relation to patient safety (Wachter and Pronovost, 2009), its definition tends to be somewhat vernacular. Political science, by contrast, has developed an extensive literature on blame that offers some helpful pointers to a more formal approach. Christopher Hood, for example, defines blame as the act of attributing something bad or wrong to some person or entity (Hood, 2011:6); it involves some (actual or perceived) harm or loss, as well as, crucially, an attribution of agency. Though he emphasises that blame is not always bad, Hood explains that, faced with external demands for accountability, blame avoidance may become a dominant preoccupation for organisations and institutions.

Many political science analyses offer a fairly muscular view of blame avoidance and blame engineering, describing a range of techniques and strategies that are purposefully chosen and implemented with specific (albeit often undeclared) intentions of deflecting or evading blame. We propose that, though much of the scrutiny has focused on the deliberate or purposeful creation of blame engineering schemes, it is possible for a system to function latently as a blame distribution and attribution system even when not designed with that goal in mind – or indeed, even, as we shall show using the example of the NHS Safety Thermometer, when it seeks explicitly to disavow a role in blaming.

### 1.2 The NHS Safety Thermometer

The declared aim of England's NHS Safety Thermometer (NHS-ST) programme is to “provide a quick and simple method for surveying patient harms and analysing results so that you can measure and monitor local improvement and harm-free care over time” (NHS Digital, 2017). The four harms measured by the NHS-ST tool – pressure ulcers, harm from falls, urinary infection in patients with catheters, and venous thromboembolism (VTE) – account for a large proportion of avoidable injury in healthcare settings, and incur high human and economic costs (Power et al., 2016). Patients who incur none of these harms are deemed “harm-free”.

The programme requires that staff caring for NHS patients England-wide in hospitals or community nursing settings (e.g. patients’ homes) record the presence and severity of the four harms on a pre-specified day each month. The NHS-ST thus creates a monthly census amounting to approximately 200,000 patients. Data collection is the responsibility of frontline teams, who are asked to record information according to the definitions in Table 1. The resulting data, which are entered into spreadsheets and aggregated at organisational, regional, and national levels, are publicly available online along with national benchmarking data. Following a 2011 pilot, the NHS-ST was introduced across England (Power et al., 2016). Since 2012/13 use of the NHS-ST has attracted financial incentives. A Commissioning for Quality and Innovation (CQUIN) payment was introduced in 2012/13 in which a financial reward was linked to data collection, with the aim of establishing a baseline. Other incentives were introduced over time; since 2015 the NHS Standard Contract has required the collection of data on a monthly basis using the NHS-ST or another local collection method.

**Table 1:** Official Guidance and Definitions for use of the NHS Safety Thermometer.

Category and definition	Values
**Age** Collected in 3 age bands	<18
	18–70
	>70
**Gender**	Male
	Female
**Old Pressure Ulcers** An ‘old’ pressure ulcer is defined as being a pressure ulcer that was present when the patient came under your care, or developed within 72 h of admission to your organisation.	None
	Category 2
	Category 3
	Category 4
**New Pressure Ulcers** New pressure ulcer developed 72 h (3 days) or more after admission to organisation. The category of the patient's worst new pressure ulcers is recorded.	None
	Category 2
	Category 3
	Category 4
**Patient Falls** Any fall that the patient has experienced within the previous 72 h in a care setting (including home if the patient is on a district nursing caseload). The severity of the fall is defined in accordance with NRLS [National Reporting and Learning System] categories.	None
	No harm
	Low harm
	Moderate harm
	Severe harm
	Death
**Catheters** An indwelling urethral urinary catheter in place at any point in the last 72 h. Record the number of days that it has been in place. If the patient has not had indwelling urethral urinary catheter in place at any point in the last 72 h, record No catheter	1-28 days
	28 + days
	Days unknown
	No catheter
**UTIs [Urinary Tract Infections]** Any patient being treated for a UTI. Record if the treatment started before the patient was admitted to your organisation (Old) or after admission to your organisation (New). Treatment for a UTI is based on clinical notes, clinical judgement and patient feedback.	No UTI
	Old UTI
	New UTI
**VTE [Venous Thromboembolism] Assessments** Is there a documented VTE Risk assessment?	No
	Yes
	N/A
**VTE Prophalyaxis** If the patient is at risk, has VTE prophylaxis started?	No
	Yes
	N/A
**VTE Treatment** If the patient is being treated for VTE choose the type of VTE. Use old VTE where the patient had the VTE before admission. Use new VTE where the patient developed the VTE after admission.	No VTE
	Old DVT [deep vein thrombosis] Old PE [pulmonary embolism]
	Old Other
	New DVT
	New PE
	New Other

### 1.3 Programme theories and institutional logics

The NHS-ST and its associated policy framework can be understood as a quality improvement (QI) programme (Portela et al., 2015). Recent years have seen growing recognition of the importance of explicating the theories or models that underlie such programmes (Davidoff et al., 2015), including elaboration of the causal assumptions – what is sometimes known as a programme's “logic”. Even when QI programmes appear to have a sound underpinning theory and logic, success is often evasive (Dixon-Woods and Martin, 2016). Many reasons for such failures can be identified, but one that has remained little examined, despite its rich explanatory potential, lies in the relationship between programme theories and institutional logics.

First proposed as a feature of institutional theory more than 25 years ago (Friedland and Alford, 1991), the literature on institutional logics has expanded greatly over the last quarter century, and now accommodates several different definitions. Broadly, however, it proposes that large-scale supraorganisational social structures tend to be characterised by distinctive sets of assumptions, values, beliefs, practices, and symbolic constructions (Friedland and Alford, 1991), offering repertoires to social actors that constrain - though do not fully determine - their choices, behaviours, and understandings. Friedland and Alford's original work focused on the central institutions of the contemporary West, such as professions, markets, and bureaucracies, but later theorists identified a hierarchical character to logics, such that “organisational fields and industries are viewed as having their own logics nested within societal level institutional orders” (Goodrick and Reay, 2011:375).

Though institutional logics contribute to the relatively stable nature of institutions over time, to the extent that they provide the organising principles for patterned behaviours, practices and symbols, they are nonetheless susceptible to alteration and transformation. Some change is linked to responses to the multiple other logics that may co-exist and potentially conflict or become more or less dominant over time (Greenwood et al., 2010); other change is linked to the agentic behaviour of individuals and organisations. However, empirically informed understanding of under what conditions a particular logic may appear most compelling and/or most directive of practice has remained limited (Martin et al., 2016). Even more poorly understood is the fate of a programme theory when introduced in an institutional field where a particular logic is especially influential.

In this article, we report a study of responses to the NHS-ST programme from multiple levels of the healthcare system. We explore the dissonances between the theory of change underlying the programme and an institutional logic of performance measurement, and in so doing show how the institutional logic displaced the programme theory's effort to eradicate blame and promote the benefits of measurement.

## 2 Methods

### 2.1 Study setting

Our study involved 19 NHS organisations in England (anonymised as sites A-S) that were participating in NHS-ST data collection and reporting. We used purposive sampling to ensure diversity of type and size as well as different levels of reported harm. Organisations were grouped into categories according to their type; we randomly selected within these. The sample included ten acute hospitals, two specialist hospitals, five community healthcare organisations, and two integrated healthcare organisations (providers of both acute and community services). Sample size was kept under review as the study progressed; 19 organisations was enough to ensure sufficient diversity.

### 2.2 Data collection

The study design was ethnographic, involving observations, interviews and documentary analysis. (Dixon-Woods and Bosk, 2010). Our model for data collection was the same in all organisations: observation of NHS-ST data collection and reporting by frontline staff on one monthly data collection day; interviews with frontline and senior staff; and collection of relevant local documentation. There were two instances in which this model could not be followed in full: at one site only NHS-ST data input and not collection was observed due to practical constraints; and at another site we did interviews only and no observation at the site's request. In total we completed ∼115 hours of observations, averaging 6.4 hours per site. Researchers were blinded to the reported rates in organisations they visited. Observers, all of whom were non-clinical researchers, took written fieldnotes and produced accounts of their visits that were transcribed in full. Two team debriefing sessions including reflection of the impact of the researchers on the data were also conducted.

As well as the ethnographic observations, we undertook interviews in the same sites with 38 senior staff with strategic responsibility for NHS-ST activity (mostly with director-level responsibility for nursing, patient safety, or QI) and 52 frontline staff (mostly ward nurses and community nurses). These interviews sought to interrogate further the approaches to data collection we had observed, and gather staff's perceptions of and attitudes towards the NHS-ST. We further interviewed four regional and national NHS leaders and 27 content experts in the four harms, comprising six with specialist expertise in VTE, five in pressure ulcers, eight in UTIs, seven in falls, and one with an overview of all four harms. We also interviewed five individuals from the NHS-ST leadership and development team, all of whom had been involved in the NHS-ST's design and/or national implementation.

The 126 interviews and all observation accounts and debriefs were audio-recorded and professionally transcribed verbatim, with transcriptions checked for accuracy against recordings. We also collected relevant national NHS-ST documentation (e.g. guidance and forms publicly available on the NHS-ST website) and local documentation (e.g. customised forms, local briefing material) at 10 sites.

### 2.3 Data analysis

A systematic and iterative approach based on the constant comparative method was used to analyse the data (Charmaz, 2006). A subset of data was initially open-coded, allowing identification of provisional thematic categories. This was followed by repeated close readings of the data to generate a thematic framework that was then tested and refined concurrently with data collection before being applied to the full dataset. Individual transcripts were compared and contrasted, and deviant cases identified and explored, in order to ensure a detailed understanding of how the NHS-ST tool was being used in different contexts. NVivo 10 software was used to support the coding, management and retrieval of data.

This project was deemed to constitute service evaluation using the criteria specified in the UK's National Research Ethics Service Guidance (Health Research Authority, 2013) and thus did not fall under the definition of research. In order to assure the ethical standing of this study, we nonetheless gained approval from a University of Leicester Committee for Research Ethics Concerning Human Subjects. Informed consent was obtained from all interview participants, and verbal permission was obtained for observations. We also followed site-specific procedures for registering service evaluations as appropriate.

## 3 Findings

We organise our findings below to show how the NHS-ST was underpinned by a programme theory that emphasised how the tool was intended to facilitate local improvement through objective, trustworthy measurement, free of the corrosive effects of blame. We show also that the organisational participants charged with using the NHS-ST tool painted a different picture: these participants saw the NHS-ST primarily as a blame allocation device, informed by their previous experiences of performance management and accountability and by institutional features of the organisation of the NHS-ST programme. Ambiguities of measurement and widespread concerns about the tool's role in allocating responsibility for harm led to concerns about fairness. As a result, participants largely saw the NHS-ST as a way not of taking the temperature of their organisations and using it to improve care, but as a way of distributing heat – the potential for blame. The reasons for this, we propose, lay in the influence of an institutional logic of accountability which displaced or overwhelmed the programme logic.

### 3.1 NHS Safety Thermometer programme theory

Interviews with the NHS-ST leadership and development team (hereafter “NHS-ST team”) about the principles underlying its development, together with analysis of official documentation and published literature, suggested that the NHS-ST could be said to have what Carol Weiss terms an *articulated programme theory* (Weiss, 1997). The goal of the programme was clear: it was to facilitate local improvement through standardised measurement across different healthcare contexts and settings in order to make visible, prioritisable, and actionable harms to patients that were otherwise obscured. The harm-free care concept - absence of all four harms - was designed to offer an alternative to surveillance of individual conditions (e.g. pressure ulcers) by focusing on the individual patient level. The NHS-ST would achieve its aims, its developers proposed, by providing the means to produce practical, straightforward, unambiguous, and locally valuable data to enable front-line teams to monitor their performance with a minimum of difficulty and to identify opportunities for improvement, free of the need to worry about attribution of blame.

*[It's about] thinking more of it as a tool within the kind of continuous improvement work that everybody wants to, to undertake and drive forward. […] So very much a kind of utilising data for improvement ethos.* (NHS-ST Team Member 5)

*The NHS ST is designed to focus on the patient and not on attribution (whose fault is it) or avoid-ability of harm. It accepts that not all harm is avoidable but works on the premise that a significant amount is and that users are working towards a goal of ‘defining the possible’ in their system. Attribution is used only as a key to system learning. The NHS ST is an attempt to shift our focus from blame to learning*. (Durkin et al., 2015)

In both interviews with the NHS-ST team and in programme documentation, achievement of the programme's goals was seen as relying on making available a neutral and trusted form of instrumentation that could “take the temperature”. The NHS-ST team described specifically seeking to avoid the issues that had challenged other NHS performance metrics, such as onerous microbiological or diagnostic criteria and complex weighting of indicators. They emphasised that they had sought to produce definitions that would be straightforward, clear and practical to use in multiple care settings and that would maximise the value and immediacy of the information for clinical teams. These considerations meant that pragmatism tended to prevail in the formulation of the definitions.

*We wanted an instrument that we could do in ten minutes per patient … If each of the harm areas [meaning content experts within them] wanted more done and more detail, we had to constantly come back to that principle.* (NHS-ST Team Member 1)

Though the tool had the potential to be used to provide comparative insights at the level of health economies, organisations and teams, the NHS-ST team was insistent that was not the intent: instead, it was to support organisations in monitoring the effectiveness of their own improvement initiatives using robust and meaningful local data, consistent with the principle that the data should not be used for blame.

*One of the things that we, and still do now, is to make clear that this isn't about comparing organisations, it's about using the information within your organisation to do quality improvement - to understand how good you are, and then to track your progress as you make changes that you hypothesise will improve the outcomes for patients, that's what it’s useful for.* (NHS-ST Team Member 4)

Many of these basic goals and assumptions of the NHS-ST programme theory were understood and accepted by some participants in our study, including NHS leaders, senior staff in organisations that collected NHS-ST data, content experts, and a few frontline staff. As proposed by the programme theory, these individuals saw measurement through the NHS-ST as enabling problems that were previously occluded to become visible, thus facilitating assessment of size and scope of quality issues, and enabling identification of targets for action and monitoring of change.

*For me, it creates awareness for us all, how [the] patient is doing … and then also it helps to improve upon some practices.* (Site L, Frontline Staff 1)

*I liked it, because it was a blunt instrument that helps staff recognise that they were harming patients and to do something about it … Putting focus on those four harms made people think about what they were doing on a day-to-day basis.* (NHS Senior Leader 3)

### 3.2 Institutional logics and the NHS Safety Thermometer as a blame allocation device

Positive views of the NHS-ST were not shared by everyone, nor was there universal acceptance of the basics of the programme theory. Identifying dissonances between the programme theory of the NHS-ST as a blame-neutral, locally-owned instrument, many organisational participants rejected a view of the tool as a straightforward, objective means of taking the temperature of safety in their organisations to support local improvement. Few frontline staff reported using the data in practice, instead interpreting the purpose of the data collection primarily as one of external reporting; the NHS-ST programme was thus seen as a form of performance management regime.

*It feels certainly that what's coming down from above is more of a ‘this is your benchmarking tool, you're not performing against other organisations in this way, what are you doing about it?’ and it's turned almost into a bit of a beast which no one can ever really sort of manage. The pressure is so great to hit all these targets, when actually the original goal of this project was to be a local improvement tool.* (Site K, Senior Staff 1)

In consequence, organisational participants characterised the NHS-ST as functioning primarily as what we term a *blame allocation device*, one that latently distributed heat – blame, shame and other unwanted consequences – in ways that they often perceived as illegitimate and unfair.

*My biggest bugbear about it is [despite] the very clear statements […] that it shouldn't be used to measure organisations or to compare organisations and that is exactly what it has done.* (Site R, Senior Staff 1)

In offering these accounts, organisational participants were interpreting the NHS-ST within an institutional, field-level logic of accountability, which they struggled to reconcile with the logic of improvement promoted by the instrument's own programme theory. This understanding was not irrational: participants drew on their experiences of previous programmes, features of the NHS-ST programme itself, and perceptions of incongruities between the espoused programme theory and what they saw as the reality of its operation in practice.

One feature of the NHS-ST that reinforced the view that it was a blame allocation device was its link to financial incentives. Though not part of the original programme theory of the developers, incentives were introduced at policy level and were accepted by the NHS-ST team as necessary to move the NHS-ST beyond the pilot phase and to scale across the NHS. In order to receive full payments linked to the NHS-ST, organisations were required to establish a baseline, identify goals for improvement and put changes into place to facilitate harm reduction, and then demonstrate “special cause variation” in the rate of the four harms that would be indicative of a change in the system over time. The use of financial incentives for QI as part of the NHS-ST meant that it tended to be understood by staff as part of a genre of performance management that they saw as oppressive and punitive.

*We had to have something like a 15% reduction in pressure ulcers, a 15% reduction in [catheter-associated] UTIs and falls and if you were over that then you would get red RAG-rated and possibly a £50,000 fine.* (Site R, Senior Staff 1)

A second feature that suggested to organisational participants that the NHS-ST was based in a logic of accountability rather than improvement was that the data were required to be reported publicly. Collation of data potentially allowed appropriation, assembly and re-assembly of data, potentially including the ranking and ordering of organisations. Though in practice the data were not compiled into “league tables”, participants in reporting organisations were highly sensitised to the possibility that they might be compared with others – to the extent that some talked about the NHS-ST as though it did engage in ranking. Senior staff in the reporting organisations thus repeatedly voiced concerns that the data could be used to make unhelpful and inaccurate comparisons between organisations, such as between community healthcare and acute hospital care, or between specialist and more general hospitals, or between particular wards. The possible use of the data by the media was seen as particularly threatening – no-one wanted their organisation to be “named and shamed.”

*We saw it as a comparison table with us and other trusts and hospitals, because it was a tool to measure against, as opposed to turning that right the way around and saying how are we failing and how can we make it better?* (Site C, Frontline Staff 1)

### 3.3 Ambiguities of measurement and ownership

The fear that the NHS-ST functioned as a blame allocation device had many of its origins in field-level logics, but as we note above, specific features of the programme itself amplified participants' perceptions. These included how the programme selected, defined, interpreted and operationalised the four harms that it aimed to measure (see Table 1). The compromises the tool's developers had made to produce operational definitions for each of the four harms had the unintended consequence of undermining participants' faith in the neutrality and objectivity of the tool, and simultaneously enhanced their perception that it was a latent source of heat. One problem was that claims of objectivity and scientific neutrality were challenged by the scientific community: in interviews, the content experts praised the principle of measurement of harm-free care, but they also argued that the individual definitions of the four harms, as deployed in the NHS-ST, did not always demonstrate clarity, completeness, and specificity. This meant that the measures did not enjoy full scientific legitimacy either among experts or among those charged with using it.

*If we're going to use those four harms we need to be absolutely clear and be completely consistent with the definition that needs to be used in the community. At the moment it is open to interpretation in my view …. So you know that would be my plea, that the definitions are really clear and that the tool was relevant to that particular setting as well.* (Site K, Senior Staff 1)

In the sites themselves, observations showed that data collectors (usually, but not always, nurses) often lacked confidence and certainty in applying the definitions. Despite extensive guidance provided on the NHS-ST website and elsewhere, substantial variability in the interpretation and application of the definitions was evident. For example, data collectors were sometimes unclear as to whether they should enter data on all urinary tract infections (UTIs), only those when a catheter was present, or only those UTIs definitively confirmed as being catheter-associated. One community-based site, contrary to guidance, only recorded falls as occurring at the patient's home if the fall happened while staff were physically present to see it: unobserved falls were not recorded. Grading the severity of pressure ulcers, which depended on the skills and experience of the data collector, was also seen as involving substantial uncertainties that were exacerbated by the linking of the measurement to financial incentives.

*Our [senior nurses] could be out there arguing the toss all the time, about is it a [grade] two or is it a [grade] three.* (Site S, Senior Staff 2)

*… you've also got the quality of the dataset it returns. People are always going to … especially if there's any form of financial penalty attached to it, people tend to start gaming to try and look as good as they can anyway. So, for instance, one of the things with pressure ulcers is that people try and reclassify them as things called moisture lesions.* (VTE Content Expert 1)

A particular focus of disquiet centred on recording recency of harms, because whether a harm was counted as “new” or “old” was critical to taking ownership of the harm and responsibility for improvement. Thus, for example, “old” pressure ulcers were defined by the guidance as those that were present on admission or that developed within 72 hours of admission, while falls were similarly defined as occurring when there was evidence of a fall in a care setting (including at home, for district nursing caseloads) in the last 72 hours. Our observations and interviews showed that the definitions were not always applied or interpreted consistently between or even within sites, in part because of reluctance to own the harms: though the programme documentation disavowed a role of “attribution” of harms, new harms were effectively ascribed to the reporting organisation for the purposes of improvement.

*[Interviewer: Do you record old pressure ulcers or falls if they happened in another care setting?] No.* (Site S, Frontline Staff 1)

*[Interviewer: So do you record all the pressure ulcers or falls that happened in another care setting?] Yes. But falls have to be within three days.* (Site S, Frontline Staff 2)

### 3.4 Fairness

Participants emphasised that *fairness* should inform the collection and use of data. Here, fairness describes the perceptions of frontline and senior staff in organisations of how far they believed that they were responsible for the harms they were being asked to record, not being blamed for the failings of others, and not being compared unjustly with others. Participants argued they should only be held accountable for harms over which there was some reasonable prospect that they might exercise agency. But they expressed substantial concerns about the fairness of the data collected by the programme and whether any comparisons that might be made across organisations would reflect individual clinical contexts. One concern was that other organisations might “game” the system in some way, leaving those who played by the rules exposed to unwarranted blame.

*We didn't want to dob anybody in it for a better word but we felt aggrieved that actually other trusts weren't doing it in the same way. This was when we found out some trusts did it over three days. Some Trusts did it after they made sure all the patients went home in the morning. None of this intelligence ever comes out when you have one of these little national conferences.* (Site R, Senior Staff 1)

A more frequent concern, however, was that the distinctive patient populations served by particular organisations made it difficult to compare them fairly with others, meaning that efforts at commensurability (transforming different qualities into a common metric) and comparability across different settings were seen as fraught with the potential for heat.

*I think sometimes it's hard to benchmark yourself against other trusts, sometimes you are measuring apples and pears and the definitions are still not clearly understood I don't think nationally, so you can be trying to compare yourself to somebody who is measuring something completely different.* (Site G, Senior Staff 1)

*For a local population, sometimes these things can have a negative impact in that people will say “ooh why is our hospital so bad” but are we comparing like with like? So for example [another local hospital] or a specialist hospital that doesn't have an A&E will have a very different profile and yet they're all in this together.* (Site H, Senior Staff 2)

A further important source of disquiet among participants in the sites centred on disputes about which organisation was responsible for reporting particular harms. Many questioned to what extent the tool, and the data derived from it, could meaningfully be used for improvement given ambiguities around where and with whom responsibility for any harm lay, and to what extent any harm was ultimately preventable. The equivocations that participants identified in determining what counted as a particular instance of a harm, its severity, and its recency could not be reported in the data themselves, which appeared tabulated in spreadsheet form devoid of contextual information. Those in the sites were concerned that these data then became available for scrutiny, judgement, and blame, without any evidence remaining of the underlying local (and social) practices involved in producing them.

*So they could come out of hospital and we [district nursing team] are not seeing them, and then we go in at 72 and a half hours and that's [deemed to be a new pressure ulcer] developed in our care. If we saw them at 71 hours that would be deemed out of our care [as an old pressure ulcer], because … [district nurse interrupts:] It would have come out of hospital.* (Site C, Frontline Staff 1 + 3)

Though intended to allow an assessment of the number of harms occurring at the level of the entire health economy, these ambiguities of attribution were deeply resented by organisational participants, who felt that they were having to taking the heat for harms that they saw as arising elsewhere or over which they had very limited control.

*I think that's what's kind of turned me off a little bit - if it's happened here, it's happened here and we should take accountability, but how can we take accountability for things that haven't happened here?* (Site H, Senior Staff 1)

*If we don't know if it was on our watch, do we class it as on our watch or not on our watch? So therefore do we class it as avoidable or unavoidable?* (Pressure Ulcer Content Expert 4)

Accordingly, the assumptions made by some organisational participants were substantially at odds with the logic promoted by the NHS-ST: rather than revealing areas for improvement and enabling action, participants saw the tool as constructing apparent failings so that they could be unfairly blamed.

*[The matron] said, “My ward is being judged by that [recorded harm]. And it's just not fair because this happened on somebody else's patch, it's just that they [the patient] happened to be on my ward at the time [of measurement]”.* (Site H, observations)

## 4 Discussion

Measurement is foundational to a safe, high quality healthcare system, but the tension between blame and accountability has proved difficult to resolve (Wachter and Pronovost, 2009, Aveling et al., 2016). A reasonable hypothesis is that QI programmes that promote low-blame approaches may encourage healthcare workers to see measurement as more facilitative of local change. This study explored a QI programme - the NHS Safety Thermometer - that sought to engage in a distinctive form of blame engineering (Hood, 2011): protecting local staff from blame in order to support local improvement to reduce patient harm. The NHS-ST was founded explicitly in a logic of measurement for improvement, and eschewed a role for blame. However, the context of its introduction was one where a dominant institutional logic is that of accountability. Influenced by the so-called New Public Management (Martin et al., 2016), with its emphasis on performance management and often harsh discipline (Bevan and Hood, 2006), it was this logic of accountability, rather than the programme theory of blame-free improvement-oriented measurement, that most strongly influenced perceptions of the NHS-ST. Frontline staff who were the target of the programme largely regarded the NHS-ST not as providing neutral, objective instrumentation that would take the temperature of safety in their organisations and support improvement, but as a latent means of exposing them to the heat of blame. Their perceptions that the NHS-ST functioned as a blame allocation device were powerfully influenced by their experiences of how the institutional logics operated in relation to other large-scale quality improvement programmes (Brewster et al., 2016). Their inferences were not irrational: while it may contain parallel and sometimes conflicting professional, patient and political narratives, the field-level logic of accountability and discipline supplied frames of reference, ways of thinking, and strongly patterned beliefs that led organisational participants to understand the programme in highly determined ways. Specific features of the programme itself, including public reporting, financial incentives, and ambiguities about definitions, appeared to embody dissonances with the programme theory and amplify the tendency for organisational participants to identify the NHS-ST as conforming to the template of performance management, rich in the potential for blame, not as the supportive and neutral measurement tool intended by its developers. Thus, though the NHS-ST has been associated with improvements in care (Buckley et al., 2014), the mechanism through which the improvement has happened is unlikely to be that of blame-free learning.

These findings have important implications for those seeking to design and implement QI programmes. In a constellation of logics (Goodrick and Reay, 2011), those underpinning a programme theory may be at risk of being outshone by others that are more established and pervasive, particularly when the programme theory is substantially misaligned with the field-level logic. This suggests that those designing QI programmes should be attentive to institutional logics, since, as Healy puts it:

*Institutions carry the criteria which people use to assess a policy's success, or the procedures for assessing alternatives to it, or the methods for implementing decisions that flow from it. Any of these may become so taken–for–granted that they appear to be the only rational way of doing things. This in turn affects the range of alternatives that may be presented as ‘realistic’ possibilities.* (Healy, 1998:63)

This may be an especially important lesson in the context of measurement in healthcare, where, in practice and policy, the arc of measurement has persistently bent towards use of data for accountability: regulators, funders, and others operate performance management systems that depend crucially on reporting of quantified data and reward (or more typically punish) accordingly. New schemes of measurement, whatever the intentions of their developers, may find it difficult to escape being categorised as more of the same. They may be especially challenged when, as in the case of the NHS-ST, features of the programme itself increase the resemblance to performance management regimes. These features may themselves be a working out of the institutional logics, suggesting that a programme theory may become distorted by the interpenetration of those logics with its own logics. Thus, for example, the NHS-ST team promoted openness in the sharing and use of data but the vehicle used for this, public reporting, is a performance instrument and was therefore misaligned with the programme theory. The potency of the public reporting mechanism may have contributed to the undermining of the claim, in the eyes of organisational participants, that the data were not for accountability. A second reason that the NHS-ST assumed the features of a performance management regime in the eyes of organisational participants was the linking of financial incentives to the establishment of robust data collection systems and to improved outcomes. The NHS-ST represents the largest data collection of its kind in the world, having collected data on around 14 million patients. It is not clear how well the NHS-ST would have succeeded in promoting a blame-free approach had it not been for these compromises in the programme theory, and this will be an important focus in the study of future QI programmes.

This study further suggests that programme designers and developers need to be highly attentive to local understandings of whether responsibility and agency are correctly and fairly attributed (Poteete, 2010) by methods of measurement (Mannion et al., 2004, Mannion et al., 2005, Dixon-Woods et al., 2014). Highly relevant to staff perceptions of the NHS-ST as a blame allocation device were the ambiguities introduced by principles intended to make the NHS-ST easy to use in multiple care settings. For many frontline workers, the data did not accomplish the objectivity, neutrality and assurances of fairness necessary to secure their confidence that unwarranted blame would be evaded. Local data collection procedures varied, sometimes significantly, and staff at all levels had reservations about the accuracy and comparability of the data. The potential for the four harm definitions of the NHS-ST to be interpreted or applied differently in different settings led to (legitimate) concerns about the commensurability of the data. These were compounded by lack of clarity about who “owned” responsibility for a particular harm, the extent to which it was avoidable, and lack of clarity about how local improvement might be driven without causal attribution of any harm recorded. In the absence of such clarity, many expressed concerns that they or their organisation might be held accountable – blamed – for harms that were unavoidable or caused elsewhere, or that inappropriate comparison with other organisations might make theirs look worse than it really was, as has also been found in other studies – for example in relation to infection control (Brewster et al., 2016) and national audit (Taylor et al., 2016).

Future efforts to design improvement programmes that reduce the risk of blame should seek to design approaches that minimise features of performance management. It may be difficult to design measurement schemes that achieve the twin goals of low burden of collection and high validity and reliability, but the risks associated with pragmatic definitions need to be managed. Further, it is clear that a sound, well-reasoned programme theory, while necessary, may not be sufficient for achieving goals for improvement in healthcare systems dominated by institutional logics that run counter to that theory. Other strategies may be helpful, for example institutional entrepreneurs, social movements, large-scale policy change, or ground-level changes in the ecology of practice (Berman, 2012). Though the NHS-ST programme did draw on some of these, maintaining the integrity of messages about a focus on ‘data for improvement’ remained challenging to achieve.

### 4.1 Strengths and limitations

This qualitative evaluation combines data from multiple sources. Our interviews with the NHS-ST developers were carried out after the programme was well into implementation and it is therefore possible they represent a rationalised account of the programme theory articulated with the benefit of hindsight. However, these interviews were not the first or only instance in which the principles underpinning the programme have been articulated: we also drew on programme documentation from past years and reports of the NHS-ST's development (Power et al., 2012, Power et al., 2016). We were able to undertake observations and interviews in a large and diverse sample of organisations, although we of course could not visit all wards or teams within these. It also proved difficult to recruit NHS senior leaders. Combining interviews with observations during site visits allowed us to both explore people's views and opinions as well as see what they actually did in practice. We were unable to assess the impact of our presence on what we observed. While only one researcher visited each organisation, we de-briefed our visits as a team – sharing observations, reflections and insights in a bid to develop a sophisticated and nuanced understanding of what we were seeing.

## 5 Conclusions

This study has lessons for those designing and implementing healthcare improvement interventions, especially in cases where the principles underpinning these run counter to, or actively seek to disrupt, established logics: it may be difficult to design interventions, measurement systems or programmes that buck wider organisational and institutional contexts. However sound a programme theory in its goals and proposed mechanisms, wider organisational and institutional conditions may frustrate its achievements. Promotion of a logic of measurement for improvement may remain challenging as long as a logic of accountability remains a dominant feature of the institutional field.

## Funding

The evaluation of the NHS Safety Thermometer was funded by NHS England (via Haelo). Natalie Armstrong is supported by a Health Foundation Improvement Science Fellowship. Mary Dixon-Woods is supported by a Wellcome Trust Senior Investigator Award (WT097899), and this award also supported Liz Brewster for this project.

## Author contributions

NA, MDW, CT and MP designed the study.

NA managed the study day-to-day.

NA, LB, CT and JW conducted observations and interviews.

LB, NA, CT, JW, RD and MDW participated in coding, analysis, and interpretation.

NA, MDW, CT and RD led drafting of the manuscript and LB, JW and MP revised it critically for important intellectual content.

All authors approved the final manuscript.
